# Causes of Delayed Diagnosis of Slipped Capital Femoral Epiphysis: The Importance of the Frog Lateral Pelvis Projection

**DOI:** 10.7759/cureus.7718

**Published:** 2020-04-18

**Authors:** Panagiotis V Samelis, Christos Loukas, Sophia Kantanoleon, Harris Lalos, Nikolaos Anoua, Panagiotis Kolovos, Flourentzos Georgiou, Apostolos-Lykourgos Konstantinou

**Affiliations:** 1 First Orthopaedic Department, Children’s General Hospital Panagiotis & Aglaia Kyriakou, Athens, GRC; 2 Orthopaedics, Orthopaedic Research and Education Center, Attikon University Hospital, Athens, GRC; 3 Orthopaedics, Children’s General Hospital Panagiotis & Aglaia Kyriakou, Athens, GRC; 4 Paediatric Orthopaedics, Orthopedic Clinic, Chania, GRC; 5 Sports Medicine, Children's General Hospital Panagiotis & Aglaia Kyriakou, Athens, GRC; 6 Orthopaedics, Katholisches Krankenhaus Dortmund-West - St. Lukas Klinikum, Düsseldorf, DEU

**Keywords:** slipped, capital, femoral, epiphysis, delayed, diagnosis, missed, iatrogenic, frog, lateral

## Abstract

Delayed diagnosis and treatment is a universally reported problem that impairs the prognosis of slipped capital femoral epiphysis (SCFE). Quite frequently, a delayed diagnosis of SCFE is observed in spite of serial admissions and examinations of the limping adolescent. Why do health professionals globally fail to make a definitive diagnosis of SCFE during the first examination of the patient? A retrospective study of 36 adolescents treated for stable SCFE and two adolescents treated for unstable SCFE has been performed. In more than half of the delayed diagnosed stable slips (13/25, 52%), the diagnosis was set after serial examinations of the patient. Health professionals commonly order only the anteroposterior (AP) X-ray view of the pelvis when examining a non-traumatic limping adolescent. The frog lateral (FL) projection is usually spared in an attempt to limit the radiation exposure of the patient, especially in ambulating adolescents with mild symptoms. It is proposed that in the non-traumatic limping adolescent, the FL projection instead of the AP pelvis view should be requested by the health professional in order to timely diagnose a surgical emergency of the adolescent hip such as SCFE.

## Introduction

Slipped capital femoral epiphysis (SCFE) is the most frequent non-traumatic cause of painful limping of adolescents, with a reported prevalence of one to 10 per 100,000 [1-2]. On microscopy, SCFE is a Salter-Harris type physeal fracture through the hypertrophic cell zone, which is the most vulnerable area (locus minoris resistentiae) of the physis [3-4]. Hormonal factors, mainly hypothyroidism (up to 40%), render the physis susceptible to shear stresses, especially in cases of concomitant obesity [1,5]. The result is abnormal movement (varus and external rotation) of the femoral neck metaphysis relative to the femoral head epiphysis [1]. In most cases, the slippage is gradual. The ambulating child complains of relatively mild symptoms such as pain and/or limp. If the duration of this first incidence of pain and limp is less than three weeks, the slip is considered an acute stable slip. After this period, new bone deposition at the posteroinferior femoral neck-head junction is evident, in an attempt of the neck periosteum to bridge the gap between the femoral head and neck, and remodeling of the anterosuperior neck initiates (chronic stable slip) [6]. Symptoms may resolve spontaneously and recur several times until diagnosis and surgical treatment are provided (acute-on-chronic stable slip). Rarely (5% of cases), an acute separation of the physis results in a dramatic clinical presentation, with extreme pain at the hip and absolute inability of the patient to walk (unstable slip) [1]. Stable slips are classified into three stages of severity, according to the Southwick - Boyer classification (slip-angle on the frog-lateral pelvis projection) [7]. Mild stable slips (slip-angle <30⁰), moderate (slip-angle 30⁰-50⁰), and severe slips (slip-angle >50⁰) [1,8].

In-situ stabilization is the widely adopted treatment for SCFE [9]. Mild SCFEs have an excellent prognosis. However, moderate and severe slips are prone to develop femoroacetabular impingement (FAI) [10-11]. Residual growth and remodeling may only partially restore the post-slip femoral neck deformity and thus may not protect from FAI. [5]. Early hip degeneration and total hip replacement, occurring 10 years earlier than expected for the general population, is the fate of the affected hip [12]. In order to deal with post-slip FAI, especially in moderate and severe slips, additional surgery, such as arthroscopic femoral neck osteochondroplasty and proximal femoral osteotomies may be necessary, either simultaneously with in situ pinning or later [10-11,13-15].

Long-term outcomes of SCFE worsen with higher slip severity [16]. On the other hand, slip severity correlates strongly with slip chronicity [16-17]. Early diagnosis and treatment is the only effective way to prevent further slippage of the capital femoral epiphysis and to obtain satisfactory long-term results with in-situ stabilization of the slipped physis [2-3,5,7-8,16-20].

The rarity of SCFE, combined with a usually mild and misleading clinical presentation, results in delayed diagnosis and treatment. When the diagnosis is finally set, the slip has usually progressed to a grade of higher severity, implying an increased risk of FAI and less favorable outcomes [5]. Delayed diagnosis of SCFE is universally reported, irrelevant of time and place [2-3,5,7-8,12,16-20]. Almost a century ago, Key reported a mean delay of 14.6 months to diagnose SCFE [3]. No real progress has been made since then towards a timely diagnosis and treatment of SCFE [2-3,5,7-8,12,16-20].

The aim of this study is to assess the impact of delayed diagnosis of SCFE on slip severity and subsequent FAI and to calculate the sensitivity of the available diagnostic signs of SCFE on the anteroposterior (AP) and frog lateral (FL) pelvis X-ray.

## Materials and methods

A retrospective study of 38 adolescents treated for unilateral SCFE from 1998 to 2011 in the Children’s General Hospital of Athens "Panagiotis & Aglaia Kyriakou" has been performed. Nineteen children were obese (mean body weight 61.2 kg), 10 had a history of endocrine disorder. Twenty-one patients were diagnosed with an acute-on-chronic stable slip, 11 patients had an acute stable slip (within three weeks of symptom onset), and four patients presented with a chronic stable slip. Two patients had unstable slips. All slips were stabilized in situ by means of Steinman pins (21 patients) or cannulated screws (15 patients). The two unstable slips were stabilized after incidental reduction using cannulated screws.

**Figure 1:**
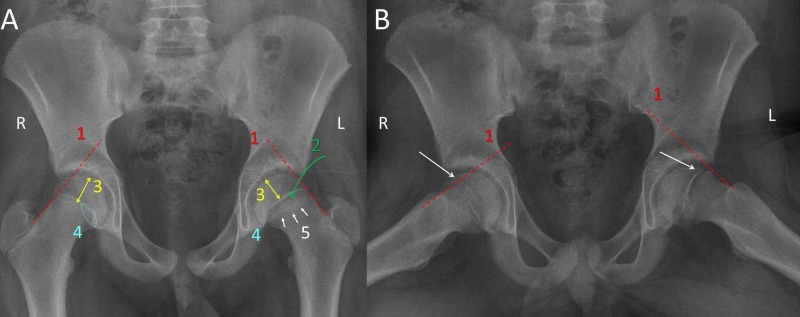
Diagnostic signs of SCFE shown on the AP (A) and FL (B) pelvis view of an 11-year-old boy with SCFE of the L hip 1. The Trethowan sign: the Klein line (red line) does not transect the capital femoral epiphysis (white arrow) of the L (SCFE) hip, as compared with the healthy R hip; 2. wide, irregular physis of the L hip; 3. decreased height of the capital epiphysis of the L hip compared to the healthy R hip; 4. the Capener sign: less overlap between the neck metaphysis and the posterior acetabular wall of the SCFE hip compared to the R healthy hip; 5. double density (multiple white arrows) of the neck metaphysis of the SCFE hip due to the overlap between the retroverted capital epiphysis and the anteverted femoral neck SCFE: slipped capital femoral epiphysis; R: right; L: left; AP: anteroposterior; FL: frog lateral

Diagnostic signs of SCFE were investigated on the AP and the FL pelvis projection, which were taken at the final diagnosis of the 36 stable slips. Radiographs from the two unstable slips were not used for measurements because extreme pain of the patients made it impossible to obtain standard projections. The Trethowan sign, that is, the absence of the transection of the capital femoral epiphysis by a line, that is the continuation of the upper or anterior femoral neck margin on the AP or FL pelvis view, respectively, (Klein line) was used as the standard diagnostic sign of SCFE [18,21]. Additional diagnostic signs of SCFE were investigated on the AP pelvis projection: an irregularly widened and blurred growth plate, the "decreased epiphyseal height sign" of the slipped epiphysis compared to the healthy contralateral hip, the Capener sign (less overlap between the posterior wall of the acetabulum and the femoral neck metaphysis) and the metaphyseal blanch sign (Steel sign: double density at the neck metaphysis due to overlapping between the posteriorly tilted capital epiphysis and the anteriorly rotated femoral neck metaphysis) [22-23] (Figure 1).

Duration of symptoms more than three weeks was used as a cut-off point between timely and delayed diagnosis and treatment. Cases that were diagnosed after serial examinations were recorded.

In five patients, subsequent contralateral SCFE developed weeks or months after the primary hip disease. In these patients, only the data of the primary slip were used since it is expected that the experience of the first hip will urge the patient to seek medical help immediately after the contralateral hip is symptomatic.

## Results

For the whole sample (n=38), the mean duration from slip onset to diagnosis was 9.6 weeks (1-32 weeks). All patients with an acute stable slip (n=11) were deemed timely diagnosed. The remaining 25 stable slips (acute on chronic, chronic) were classified as the delayed diagnosis group.

The 36 patients with a stable slip were allocated according to slip severity for timely (≤3 weeks) or delayed (>3 weeks) treatment (Table 1).

**Table 1:** Slip severity for timely (≤ 3 weeks) and delayed (>3 weeks ) diagnosis of SCFE SCFE: slipped capital femoral epiphysis

Duration of symptoms from onset to diagnosis of SCFE	Slip severity (slip angle)			
	n	mild <30º	moderate 30-50º	severe >50º
≤ 3 weeks (timely diagnosis)	11	7	4	0
	%	63.6	36.4	0.0
>3 weeks (delayed diagnosis)	25	13	9	3
	%	52.0	36.0	12.0
Total	36	20	13	3
	%	54.5	36.4	9.1

Signs of FAI (subchondral sclerosis, intraarticular space narrowing and bone spurs) were assessed on radiographs obtained at physis fusion and implant removal (seven to 37 months after index surgery, mean: 14.3 months) (Table 2).

**Table 2:** Frequency of FAI-related signs at implant removal for timely (≤ 3 weeks) and delayed (>3 weeks ) diagnosis of SCFE SCFE: slipped capital femoral epiphysis, FAI: femoroacetabular impingement

Duration of symptoms from onset to diagnosis of SCFE	Number of hips	FAI signs at implant removal
	36	21
	%	58.3
≤ 3 weeks (timely diagnosis)	11	6
	%	54.5
> 3 weeks (delayed diagnosis)	25	15
	%	71.4

All patients had, by definition, a positive Trethowan sign on FL pelvis view (100%). The AP view was less efficient to present all diagnostic signs of SCFE: 28 patients (77.8%) had a widened growth plate, only 18 patients (50%) presented a positive Trethowan sign, a diminished height of the capital epiphysis and the Capener sign were positive in 17 (47.2%) patients, and only five patients (13.9%) had a pathologic metaphyseal blanch sign (Table 3).

**Table 3:** Frequency of the radiologic signs of SCFE on the AP and FL pelvis view SCFE: slipped capital femoral epiphysis; AP: anteroposterior; FL: frog lateral

	FL view	AP view				
	Trethowan sign (Klein line)	Wide, irregular physis	Trethowan sign (Klein line)	Decreased epiphyseal height sign	Capener sign	Metaphyseal blanch (Steel) sign
n	36	28	18	17	17	5
%	100	77.,8	50.0	47.2	47.2	13.9

Thirteen patients (52%, 13/25) of the delayed diagnosis group and the two unstable slips had at least one medical examination (primary care provider, orthopedic surgeon, resident, radiologist) before the diagnosis was set. In most cases, the patients were examined by a non-orthopedic. On the first admission, four patients did not have any radiologic examination, one patient had an X-ray of the lumbar spine and one had an X-ray of the ipsilateral knee. Another patient had an X-ray of the ipsilateral femur and was treated with a long limb cast for two weeks. Three patients underwent a radiologic examination of the pelvis (pelvis AP and FL projection) on the first medical examination, but both projections were deemed negative for a fracture. The symptoms were attributed to sports or were thought to be pain due to growth and the patients were advised to rest. In total, only six patients (6/15, 40%) had an FL pelvis view on the first examination but, still, no diagnosis was set. Rest was recommended in one of the two unstable slips two weeks prior to the slip. For the other patient with an unstable slip, the physician suspected a pre-slip SCFE after inspection of the AP pelvis view but ordered an MRI of the hip, which was never performed because the patient had an acute unstable epiphysiolysis the next day. Collectively, the mean delay until diagnosis in these patients was 9.21 weeks (one to 32 weeks), which is similar to the mean delay of the whole group of patients.

## Discussion

Studies report a mean delay in the diagnosis of SCFE of about five to 10 months but extremes of up to three years from onset to diagnosis have also been reported [2-3,16-20]. A greater delay results in a slip of higher severity and worse long-term outcomes after treatment [2-3,7-8,12,14,16-20,24-25]. Kocher found a significant relationship between a delay in diagnosis and slip severity (<30⁰: 10 weeks, 30⁰-50⁰: 14.4 weeks, >50⁰: 20.6 weeks) [17]. Slip severity increases approximately by one level for each month of delay [2,17].

The etiology of delayed diagnosis of SCFE is multifactorial. A delayed diagnosis of SCFE may be patient-related (late admission) or physician-related. The former encompasses all causes (the patient’s personal perception of pain and limp, financial-, social-, geographical-, family-related issues, availability and accessibility of health services, insurance status of the patient) that may hinder timely medical examination, and, hence, early diagnosis and treatment of the limping child. The latter includes delayed diagnosis after examination of the patient by a health professional.

The hip disease may not be suspected. In only half of the cases, the patients locate the pain at the hip joint [16]. Pain may reflect on the ipsilateral thigh and knee, or the patient may just complain of a painless limp [16,24-26]. Radiographs may not be requested, or only the AP pelvis projection will be ordered, in an attempt to spare unnecessary radiation exposure of the child. On the other hand, a negative initial examination does not always result in a delay of diagnosis or treatment, if the patient seeks a repeat medical examination soon after the first. Five out of the 13 stable slips that were not diagnosed on the first examination were timely diagnosed within the first three weeks from the onset because the patients were reexamined within a few days.

The AP pelvis projection may not be diagnostic of SCFE. Klein described the FL pelvis projection in 1952 [18]. The importance of this projection was further evaluated by several authors, all of whom stated that the AP pelvis view has low sensitivity to detect minor slips. Cowell found that the sensitivity of the AP pelvis view drops from 86% in unilateral slips to 64% in bilateral cases while the FL view gives a positive diagnosis in 100% of cases [16]. Green et al. quantified the extent of transection of the capital femoral epiphysis by the Klein line, aiming to increase the sensitivity of the AP pelvis view to diagnose a slip. Thus, a greater than 2 mm side-to-side difference of the transection of the capital epiphysis by the Klein line increases the sensitivity of the AP pelvis view to detect an SCFE to 79% [27]. Song et al. showed that the sensitivity of the AP pelvis view to diagnose a slip increases to 76% if the acetabulotrochanteric distance is greater than 2mm [19]. Bloomberg et al. found that a wide, irregular physis is the most reliable sign of an early slip on the AP pelvis projection. The AP pelvis view was ineffective to provide a diagnosis in 11% of cases and the authors recommend a routine lateral radiograph when SCFE is suspected. Our study is consistent with this finding since we found a 77.8% sensitivity of the wide-irregular-physis sign to detect SCFE on the AP pelvis view [23]. Bomer et al., on the other hand, showed that a solitary FL pelvis view is equally efficient to the combination of AP and FL to diagnose painful hip pathology of the child, such as SCFE and Legg-Calve-Perthes disease as well, and recommend that only the FL should be requested in such cases [28].

Non-orthopedics are more prone not to diagnose a slip during the first examination of the patient [29]. Physicians must be aware of the rare conditions of the hip that may cause a limp. The absence of trauma in addition to the patient's age should alert the physician not to rely only on history and clinical examination but also to proceed to appropriate radiologic control of the pelvis, including the FL pelvis view [30].

The present study agrees with published knowledge: The mean delay until the diagnosis of SCFE was 9.6 weeks for the whole SCFE sample. It is also confirmed that a longer delay of SCFE diagnosis is associated with slips of higher severity and hence of a higher risk for FAI. Thus, severe slips (three patients) were diagnosed only in the delayed diagnosis group but only one of the three had more than one medical examination until the diagnosis of SCFE. The radiologic signs of FAI were more frequently manifested in the delayed diagnosis group (15/25 patients of the delayed diagnosis group compared to 6/11 patients in the early diagnosis group). This difference was not significant (chi-square, p>.05), probably, because of the small sample size or because mild slips are not spared from FAI as well (Table 2) [11]. Furthermore, this study confirms that solely the FL pelvis view - and not the AP view - is safe to diagnose SCFE (Table 3). Therefore, the FL view should be ordered first, prior to the AP view, when examining a limping adolescent.

This study has certain limitations. As any retrospective case series, it has a selection bias, because it includes patients who were referred to the hospital by other physicians and patients who were first-time admissions. Patients with incomplete records were excluded from the study. Thus, the ratio between the various clinical presentations of SCFE according to slip chronicity (acute, acute on chronic, chronic) or severity (mild, moderate, severe) that was calculated in this study might not represent the general population.

## Conclusions

SCFE should be a key component of the differential diagnosis of every non-traumatic limp of the adolescent. The prompt diagnosis is the only controllable factor in order to improve long-term outcomes with in situ slip stabilization and avoid additional surgery such as hip arthroscopy or proximal femoral osteotomy. The frog lateral projection sets the diagnosis of SCFE in 100% of cases, irrelevant of slip severity, and should be always requested by the health professional, in order to diagnose the non-traumatic pathology of the limping adolescent early.
